# The Gut Microbiota in Young Adults with High-Functioning Autism Spectrum Disorder and Its Performance as Diagnostic Biomarkers

**DOI:** 10.3390/nu17111748

**Published:** 2025-05-22

**Authors:** Jiangbo Ying, Xinran Xu, Ruwen Zhou, Arthur C. K. Chung, Siu Kin Ng, Xiuyi Fan, Mythily Subramaniam, Sunny H. Wong

**Affiliations:** 1 Department of Developmental Psychiatry & East Region, Institute of Mental Health, Singapore 539747, Singapore; jiangbo_john_ying@imh.com.sg; 2 Lee Kong Chian School of Medicine, Nanyang Technological University, Singapore 308232, Singapore; xinran007@e.ntu.edu.sg (X.X.); ruwen001@e.ntu.edu.sg (R.Z.); arthur.chungck@ntu.edu.sg (A.C.K.C.); siukin.ng@ntu.edu.sg (S.K.N.); xyfan@ntu.edu.sg (X.F.); 3 Research Division, Institute of Mental Health, Singapore 539747, Singapore; 4 Department of Gastroenterology and Hepatology, Tan Tock Sen Hospital, Singapore 308433, Singapore

**Keywords:** gut microbiota, autism, adult, biomarker, diagnostic tool

## Abstract

**Background/Objectives:** Diagnosing ASD in adults presents unique challenges, and there are currently no specific biomarkers for this condition. Most existing studies on the gut microbiota in ASD are conducted in children; however, the composition of the gut microbiota in children differs significantly from that of adults. This study aimed to study the gut microbiota of young adults with high-functioning ASD. **Methods:** Using metagenomic sequencing, we evaluated the gut microbiota in 45 adults with high-functioning ASD and 45 matched healthy controls. **Results:** Adjusting for sociodemographic information, dietary habits, and clinical data, we observed a distinct microbiota profile of adults with ASD in comparison to controls, with the intensity of autistic traits strongly correlating to microbial diversity (correlation coefficient = −0.351, *p*-value < 0.001). Despite a similar dietary pattern, the ASD group exhibited more gastrointestinal symptoms than the healthy controls. An internally validated machine-learning predictive model that combines the Autism Spectrum Quotient questionnaire score of individuals with their microbial features could achieve an area under the receiver operating characteristic curve (AUC) of 0.955 in diagnosing ASD in adults. **Conclusions:** This study evaluates the gut microbiota in adult ASD and highlights its potential as a non-invasive biomarker to enhance the diagnosis of ASD in this population group.

## 1. Introduction

Autism spectrum disorder (ASD) is a complex neurodevelopmental condition characterized by difficulties in social communication and interaction, as well as the manifestation of repetitive and restrictive patterns of behavior, activities, or interests [1]. Globally, the prevalence of ASD has been reported to range from 0.01% to 4.36%, with a median prevalence of 1% [2]. In the United States, it has been estimated recently that the overall ASD prevalence is 2.76% (one in 36 children), which is higher than previous estimates made from 2000 to 2018 [3]. ASD is associated with a considerable impact and economic burden on families, as well as a burden on public health. For example, the lifetime cost for an individual with ASD in China is USD 2.65 million for those without intellectual disability (ID) and USD 4.61 million for those with comorbid ID [4].

The exact pathogenesis of ASD is still not understood, although it is widely considered to result from the interactions between genetic and environmental factors [5]. Hundreds of genes have been identified to contribute to the significant deficits in social cognition and communication in ASD, but these genetic factors account for only 10–20% of ASD cases [6]. Various environmental factors, such as gestational diabetes and a low birth weight, are reported to be associated with ASD [7]. The gut microbiota has recently been identified to play an important role in the gut–brain axis and may contribute to the development of ASD. The gut–brain axis involves a bidirectional connection with the central nervous system, and occurs through several mechanisms, such as the autonomic nervous system, the enteric nervous system, neurotransmitters, and hormones [8]. Various studies have observed an altered gut microbiota composition in persons with ASD [9,10,11]. These observations are supported by interventional studies, where the transfer of fecal microbiota from ASD donors into germ-free mice has been shown to induce autistic behaviors [12]. In humans, microbiota transfer therapy has been found to mitigate symptoms of ASD [13]. In addition, a recent study has demonstrated strong correlations between ASD symptoms and urinary metabolites, such as 3-hydroxyhippuric acid and homovanillic acid, following fecal microbiota transplantation [14]. These findings further implicate the gut microbiota in the metabolic disturbances associated with ASD.

Most studies on the gut microbiota to date have focused on children with ASD. A recent systematic review in this area reveals that only one study has assessed the microbiota in adult individuals with ASD, and this study only examined the bacterial microbiota [9]. A recent study has applied metagenomic sequencing to study multi-kingdom microbiota, but only included children aged 1–13 years [15]. Since the gut microbiota is correlated with age [16], the differences in the composition of gut microorganisms between children and adults may not be consistent.

Adults with ASD exhibit different clinical presentations compared to children. Individuals with ASD who do not have intellectual impairment are considered as having high-functioning ASD [17] or ASD without accompanying intellectual impairment [1]. Although ASD traits usually start in the early developmental period, symptoms may not become fully apparent until adulthood for high-functioning ASD [18]. Diagnosing ASD especially high-functioning ASD, in adult individuals, presents several challenges, such as the inaccurate recall of childhood history, behavioral overlap with other mental disorders, and learned coping strategies to mask certain ASD symptoms [19]. There are currently no reliable biomarkers to effectively diagnose ASD in adults, and sometimes ASD among adults can even be misdiagnosed as other psychiatric conditions [20]. Therefore, it is worthwhile to explore the gut microbiota as potential biomarkers for diagnosing ASD among adults.

The present study aimed to comprehensively investigate the gut microbiome and its functional pathways among young adults with high-functioning ASD and evaluate their potential application as a diagnostic tool for adults with ASD. We hypothesized that adults with ASD would have an altered microbial composition, compared to the healthy controls, and that the observed different microbial compositions could facilitate ASD diagnosis in adults.

## 2. Methods

### 2.1. Study Participants

Persons with ASD aged between 21 and 40 years old were recruited from the Adult Neurodevelopmental Service (ANDS) at the Institute of Mental Health (IMH). The IMH is the only tertiary psychiatric hospital in Singapore. The ANDS provides both inpatient and outpatient services for adults with ASD and/or ID, and its new case clinic has the capacity to review 319 patients over a two-year period [21]. The diagnosis of high-functioning ASD was made by qualified psychiatrists according to the criteria outlined in the Diagnostic and Statistical Manual of Mental Disorders, Fifth Edition (DSM-5), for ASD without intellectual or language impairment [1]. Healthy controls with similar gender, age range, and ethnicity were recruited from the community, and they were required to have no self-reported history of ASD or other mental health conditions. All participants were recruited from October 2023 to April 2024. Recruitment efforts included distributing flyers and advertisements targeted at hospital staff to encourage referrals and participant enrollment. Written informed consent was obtained from all participants. All study procedures were performed in accordance with the relevant laws and institutional guidelines and were approved by the National Healthcare Group Domain Specific Review Board.

Exclusion criteria for both ASD group and control group included having intellectual disability; receiving any formulated probiotic or prebiotic supplements; and being treated with any antibiotics within one month of the study.

### 2.2. Sample Size

The sample size was calculated a priori using G* Power software (version 3.1.9.6) [22]. In a previous study exploring the relationship between gut microbiota and autism [10], the alpha diversity index, estimated by the Chao1 index, was 79.8 (standard deviation, SD = 19.3) in the ASD group and 92.6 (SD = 20.2) in the control group. Based on the differences in the ecological index obtained by this study, 41 participants in the ASD group and another 41 in the control group were needed to achieve a power of 80% with an alpha error of 0.05. Assuming a drop-out rate of 10%, the present study aimed to recruit 45 participants in each group.

### 2.3. Questionnaires

Demographics and clinical information: social demographics, including age, gender, ethnicity, body mass index (BMI), marital status, education level, and monthly household income, were collected for both ASD group and control group.

The Autism Spectrum Quotient was used to quantify ASD traits in participants. The Autism Spectrum Quotient is a widely used tool in clinical practice and research to quantify ASD symptoms [23]. It has 50 items with a maximum score of 50. It has an acceptably high sensitivity and specificity. At a cut-off score of 26, it has a sensitivity of 0.95, specificity of 0.52, positive predictive value of 0.84, and negative predictive value of 0.78 [24]. The questionnaire has been used in Asian countries, such as Japan [25].

The Functional Gastrointestinal Checklist (FGI Checklist) was used to assess gastrointestinal symptoms of participants. The FGI checklist was initially developed to measure both upper and lower gastrointestinal symptoms [26]. It assesses 20 common gastrointestinal symptoms, such as regurgitating, heartburn, epigastric pain, diarrhea, and abdominal distension.

The Diet Screener questionnaire was used in this study to explore the dietary habits of participants. This 37-item questionnaire was developed to assess dietary patterns in a multi-ethnic population in Singapore [27]. It evaluates the average food intake of 37 food/beverage items over the preceding year. Each item is rated on a 10-point scale ranging from “never or rarely” to “6 or more times a day”. The items are categorized into the following seven groups: vegetables, fruit, nuts/legumes, whole grains, low fat dairy, red and processed meat, and sweetened beverages. Based on the scores obtained in each of the seven groups, each participant was assigned a score between 1 and 5 corresponding to the intake quintile they belong to, with reverse scoring applied for meat and sweetened beverages. Subsequently, these seven quintile scores were added together to calculate the total value, which is the Dietary Approaches to Stop Hypertension (DASH) score. A detailed description on the calculation of the DASH score can be found in a previous study [28].

### 2.4. Sample Collection, DNA Extraction and Sequencing

Approximately 200–300 mg of stool samples were collected from each participant using a stool collection kit. Preservative media (nuclei acid preservative, cat: 28330, Norgen Biotek Corp., Thorold, ON, Canada) were included in the stool collection tubes to allow safe transportation of microbial DNA at room temperature. Stool samples in preservative media were delivered to the laboratory to be stored at −80 °C refrigerators until further processing. DNA was extracted from the stool samples with the QIAamp PowerFecal Pro DNA Kit (QIAGEN, Hilden, Germany), following the manufacturer’s instructions. The DNA concentration and A260/280 ratio were measured with a NanoDrop Spectrophotometer for quality control. The extracted DNA was then sent for shotgun metagenomic sequencing on the Illumina NovSeq 6000 platform (Novogene, Singapore).

### 2.5. Bioinformatics and Statistical Analysis

Raw sequencing reads were first quality filtered and trimmed using Trimmomatic ver 0.39 [29]. To remove host-associated reads, filtered reads were mapped against the human reference genome (hg38) using bowtie version 2.4.5 [30]. Read pairs with any read mate that was aligned to the reference genome were discarded for further analysis. To profile the distribution of microbial species, the taxonomic classification of cleaned reads resolved at strain level was carried out using MetaPhlAn version 4 [31], a marker-gene-based metagenomic taxonomic profiler that is based on the marker genes identified from over one million prokaryotic and metagenome-assembled genomes. The profiles of microbial functional pathways (e.g., MetaCyc, KO gene family) were obtained using HUMAnN ver3.0 [32]. To account for the difference in library size, mapped read counts were converted into copies per million (CPM) values prior to downstream analyses. For the abundance estimation of fungi and viruses, cleaned reads were mapped against the NCBI Fungi and refseq viral databases, respectively, using Kraken2 version 2.1 [33] after removing bacterial reads by mapping cleaned reads against the unified human gastrointestinal genome (UHGG version 2.1) database [34].

Sequencing data were analyzed using R (version 4.4.1). Alpha diversity metrics, including Simpson, Shannon, and Chao indices, were calculated with the “vegan” package [35]. Beta diversity was assessed using PERMANOVA (adonis) in the “vegan” package. Both alpha and beta diversity analyses were adjusted for age, gender, ethnicity, BMI, FGI Checklist score, and DASH score. The differences in microbiota abundance and functions between the ASD and control groups were evaluated by the “phyloseq” [36] and “DESeq2” packages [37]. Heatmap plots were generated using the “pheatmap” package and volcano plots were created by the “ggplot2” and “ggrepel” packages. Network plots were generated using the “igraph”, “ggraph”, and “reshape2” packages. Corrections for multiple hypothesis testing were applied using the false discovery rate (FDR) approach.

The random forest model in Python (version 3.8.19) was used to identify specific microbial taxa and functions that were different between the ASD and control groups. Receiver operating characteristic (ROC) curve analysis was performed to assess the predictive power of the biomarkers selected from the model, followed by calculation of the area under the curve (AUC). Mean AUC value based on 100 performances was calculated to provide a robust estimate of the model’s predictive capability.

## 3. Results

### 3.1. Demographics and Clinical Characteristics of the Participants

A total of 45 individuals with ASD and 45 control subjects were recruited for this study. The two groups were comparable in terms of their gender and BMI distributions. In the ASD group, there were 41 males and 4 females, with a median age of 23 years (interquartile range [IQR] = 4) and a median BMI of 26.0 kg/m^2^ (IQR = 8.77). The control group included 40 males and 5 females, with a median age of 25 years (IQR = 4) and a median BMI of 24.5 kg/m^2^ (IQR = 6.23). Most participants were ethnic Chinese and resided in government flats. The demographic information is summarized in Table 1.

The ASD group scored higher on the Autism Spectrum Quotient questionnaire (median = 30, IQR = 10) than the control group (median = 18, IQR = 11, *p*-value < 0.001), indicating greater autistic traits in the ASD group. The FGI Checklist scores were also higher in the ASD group (median = 4, IQR = 8) than the control group (median = 2, IQR = 4, *p*-value < 0.001), revealing more gastrointestinal symptoms among the participants with ASD. However, the DASH scores were similar between the ASD group (mean = 20.53, SD = 3.68) and the control group (mean = 20.53, SD = 4.65), with no significant differences being observed in the intake of the seven food groups that were evaluated. The Diet Screener Questionnaire also indicated that none of the participants were following a special diet, such as a vegetarian diet.

### 3.2. Gut Microbial Diversity Between ASD and Control Groups

**Figure 1:**
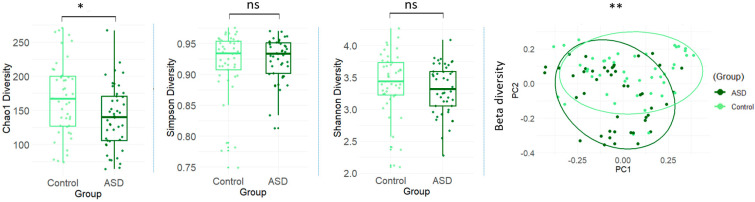
Gut microbial diversity between ASD and control groups. Alpha diversity indices at species level measured by Chao1 index (*p* = 0.025), Simpson index (*p* = 0.335), and Shannon index (*p* = 0.548). Beta diversity measured by principal coordinate analysis of the Bray–Curtis distance. Each point represents samples and the circles surrounding the points indicate 80% confidence interval. *p*-values were adjusted for age, gender, ethnicity, BMI, FGI Checklist score, and DASH score. **: *p*-value < 0.01, *: *p*-value < 0.05, ns: *p*-value ≥ 0.05 or not significant.

A total of 993 bacterial species were identified across all participants. Alpha diversity analysis showed that the within-subject microbial richness was significantly lower in the ASD group than in the control group, as indicated by the Chao1 index (140 ± 46.1 (mean ± SD) in the ASD group and 168 ± 54.8 in the control group, *p* = 0.025), although the Simpson (0.925 ± 0.0341 in the ASD group and 0.918 ± 0.0531 in the control group, *p* = 0.335) and Shannon (3.31 ± 0.390 in the ASD group and 3.40 ± 0.503 in the control group, *p* = 0.548) indices did not show significant differences (Figure 1). Furthermore, the Autism Spectrum Quotient scores were significantly correlated with the Chao1 index (correlation coefficient = −0.351, *p*-value < 0.001) (Figure S1 in the Supplementary Material), suggesting that the individuals with more ASD symptoms had lower microbial richness. Beta diversity analysis revealed that the microbial composition differed significantly between the two groups, as shown by principal coordinate analysis (PCoA) based on the Bray–Curtis distances (*p* = 0.001; Figure 1). The PCoA was performed using a Bray–Curtis distance matrix calculated from species-level relative abundance data from across all samples. The two principal coordinates, PC1 and PC2, explained 14.14% and 8.68% of the total variation in beta diversity, respectively.

### 3.3. Abundance of Microbial Species Between ASD and Control Groups

**Figure 2:**
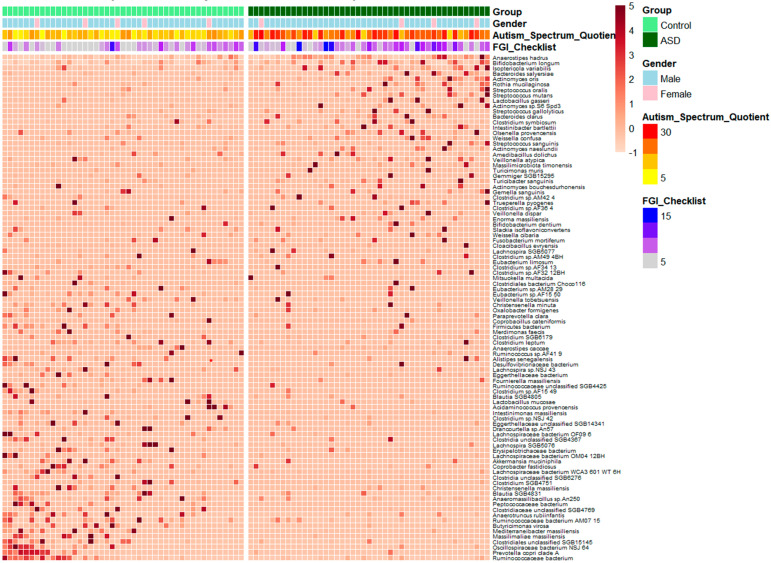
Heatmap depicts abundances of 94 bacterial species which appeared in ≥10% of samples and had distinct profiles between ASD and control groups.

**Figure 3:**
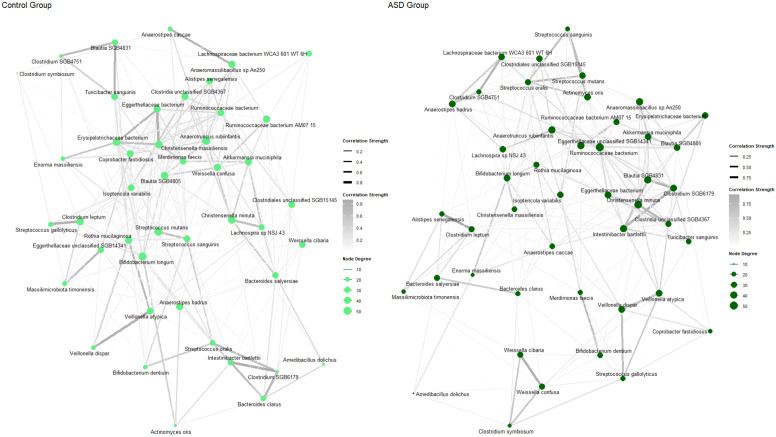
Network visualization of bacterial species which appeared in ≥15% of samples in the Control group and ASD group. Node color represents group and node size (degree) indicates the number of direct interactions for a given microbe. Color depth and width of each line reflect the strength of interaction (edge weight).

Of the 993 bacterial species that were identified, 94 were present in ≥10% of the samples and exhibited distinct profiles between the ASD and control groups (Figure 2). Species such as *Anaerostipes hadrus*, *Lactobacillus gasseri*, *Weissella confusa*, and *Eubacterium limosum* were enriched in the ASD group. In contrast, species like *Prevotella copri clade A* and *Ruminococcaceae bacterium* were more prevalent in the control group. Furthermore, several microbial species, such as *Anaerostipes hadrus* and *Actinomyces naeslundii*, showed increased abundance in certain individuals within the ASD group who had higher Autism Spectrum Quotient scores, although consistent patterns were not observed. There were no clear patterns between the distribution of bacterial species and the distribution of gastrointestinal symptoms, but overall, the gastrointestinal symptoms, measured by the FGI Checklist scores, were more severe in the ASD group (Figure 2). The interactions between the bacterial species in the ASD group and those in the control group are presented in Figure 3. The ASD group exhibited a distinct microbial interaction pattern when compared to the control group. The top 50 bacterial species with the largest log2 fold changes are highlighted in the volcano plot in Figure S2 of the Supplementary Material.

Across all samples, a total of 50 fungal species were identified. Notably, species like *Meyerozyma caribbica* and *Mucor circinelloides* were more prevalent in the ASD group, whereas *Dictyocoela roeselum* and *Podosphaera cerasi* were more abundant in the control group (Figure S3). A total of 40 viral species were found across all samples. Some viruses, such as *Carjivirus hominis* and *Oengusvirus oengus*, were more abundant in the ASD group. In contrast, viruses like *Aurodevirus hiberniae* and *Toutatisvirus toutatis* were more commonly seen in the control group. Figure S4 illustrates the differences in the distribution of viral species between the ASD and control groups.

Among the 174 MetaCyc pathways observed in all of the samples, 24 differential pathways were identified. Pathways such as the inosine-5′-phosphate biosynthesis III (PWY-7234) and thiamine diphosphate salvage IV (PWY-7356) pathways were positively associated with ASD, while pathways such as the pyrimidine ribonucleotides de novo biosynthesis (PWY0-162) and geranylgeranyl diphosphate biosynthesis II (via MEP) (PWY-5121) pathways showed negative associations with ASD (Figure S5 in the Supplementary Material).

### 3.4. Microbial Biomarkers for ASD Diagnosis

The potential of using microbial biomarkers for ASD diagnosis was investigated using a random forest model. To ensure the robustness and reliability of the evaluation, we adopted a repeated holdout validation strategy rather than a single split strategy. Specifically, the dataset was randomly partitioned into 70% training and 30% test sets, and this splitting procedure was repeated independently 100 times to account for variability in the data selection. Within each iteration, three-fold cross-validation was conducted on the training set to select optimal model hyperparameters. After tuning, the model was retrained on the entire training subset using the selected parameters and evaluated on the corresponding test set. Finally, the classification metric AUC was averaged across the 100 runs to report the model’s overall performance. This repeated evaluation strategy provided a comprehensive and statistically stable estimate of the model’s predictive power.

**Figure 4:**
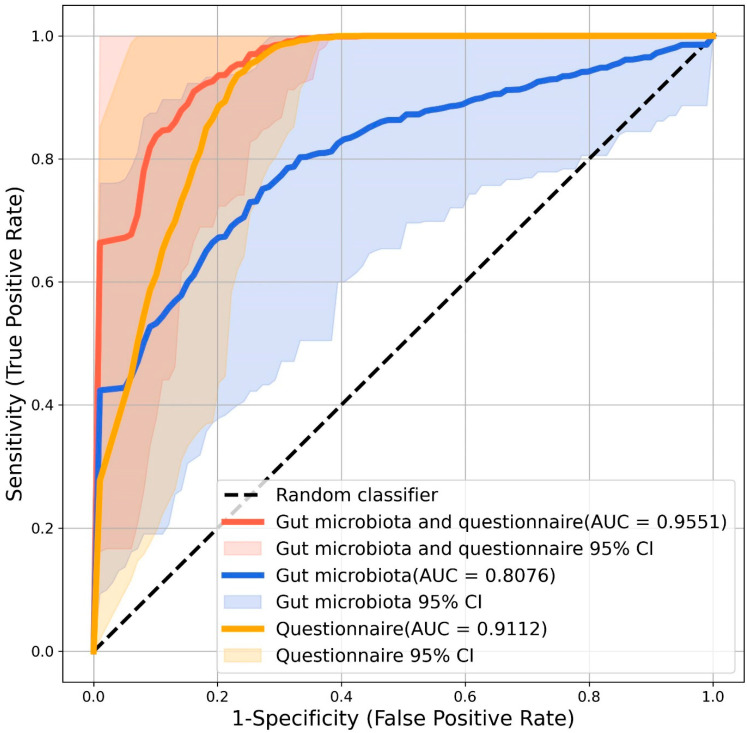
Random forest performance analysis. The ROC curves were generated based on three datasets: gut microbiota data combined with Autism Spectrum Quotient questionnaire data (red curve), gut microbiota data alone (blue curve), and Autism Spectrum Quotient questionnaire data alone (yellow curve).

The associations between the number of features selected and the mean AUC values are presented in Figure S6 in the Supplementary Material. To achieve a good balance between the AUC values and the number of features, a total of 25 optimal features (Table S1 and Figure S7 of the Supplementary Material), including 20 bacterial species, 3 fungal species, and 2 microbial pathways, were included in the prediction model. When only these 25 microbial markers were included, the model achieved a mean AUC of 0.8076. In contrast, when only the autism spectrum quotient scores were used, the mean AUC was 0.9112. Notably, by combining both the microbiota markers and the Autism Spectrum Quotient scores, the mean AUC improved to 0.9551 (Figure 4). To provide a more comprehensive understanding of the model’s performance, we evaluated additional classifiers, including logistic regression, decision tree, and naïve Bayes classifiers, using the same dataset and evaluation metrics (Figures S8–S10 in the Supplementary Material). While the random forest model performed similarly to or even better than the other models in terms of its overall accuracy and AUC, the differences in performance varied depending on the specific model and data input.

To better illustrate the performance of each model, we compared the classification outcomes of the models based solely on the gut microbiota, the Autism Spectrum Quotient questionnaire data, and their combination (see Table S2 in the Supplementary Material). This comparison shows that the questionnaire-based model had high sensitivity, while the gut microbiota-based model demonstrated more balanced true positive and true negative rates. Combining both data sources did not significantly improve the overall classification accuracy but retained the high sensitivity observed in the questionnaire-based model.

## 4. Discussion

To the best of our knowledge, this is the first metagenomic study to explore the gut microbiota in young adults with high-functioning ASD. Our findings demonstrate that the microbiota composition in adults with ASD is significantly different from that of healthy controls, with the richness of the bacterial species being strongly correlated with the intensity of ASD symptoms. The microbial features in the gut microbiota, including bacteria, fungi, viruses, and functional pathways, could be useful in identifying adults with ASD.

In this study, certain bacterial species differed between the ASD and control groups. Although many of these species have not been consistently reported across previous studies, some of our findings align with research conducted in children. For example, the ASD group showed a reduced abundance of *Prevotella copri* and an increased abundance of *Eubacterium limosum*, which is consistent with findings from earlier pediatric ASD studies [38,39]. *Prevotella copri*, a predominant species in the *Prevotella* genus, is commonly found in the human gastrointestinal tract [40] and generates short-chain fatty acids (SCFAs) that protect the mucosal barrier and reduce the risk of inflammation [41]. In contrast, although *Eubacterium limosum* has been reported to ameliorate colonic inflammation, its metabolic role in the gastrointestinal tract remains poorly understood [42]. We also found that several bacteria in the *Ruminococcaceae* family, such as *Ruminococcaceae bacterium* and *Ruminococcaceae bacterium AM07-15*, were reduced in abundance in the ASD group. Our results align with another study in children which showed an inverse association between the abundance of butyrate-producing *Ruminococcaceae* and ASD [43]. SCFAs, which include acetate, butyrate, and propionate, are key bacterial metabolites that mediate communication along the microbiota–gut–brain axis [44]. They are primarily produced through the fermentation of dietary fiber by gut microbes such as *Prevotella* and *Ruminococcus* [45]. Alterations in the gut microbiota may disrupt this bidirectional regulatory pathway between the gut and the brain [46]. SCFAs play a significant role in neuroimmune regulation by crossing the blood–brain barrier or by activating sensory neurons of the vagus nerve [47]. A deficiency in SCFA-producing bacteria has been associated with several psychiatric and neurological disorders, including ASD, anxiety, and Parkinson’s disease [48].

Interestingly, this study revealed that several bacteria with potential probiotic properties, such as *Anaerostipes hadrus*, *Weissella confusa*, and *Lactobacillus gasseri*, were increased in the ASD group. To the best of our knowledge, these findings have not been reported in other studies. *Anaerostipes hadrus* is known for its butyrate producing capacity and contains biotin synthesis genes that could regulate inflammation and immunity [49], making it a potentially beneficial microorganism [50]. However, it is also reported that *Anaerostipes hadrus* can affect the availability of long-chain free fatty acids in the portal circulation, contributing to hepatic fibrosis [51]. *Weissella* confusa, often isolated from fermented foods, has been proposed as a potential probiotic [52,53]. The probiotic *Lactobacillus gasseri* can offer various health benefits, including the maintenance of gut homeostasis and immunomodulation, as supported by genomic and empirical evidence [54]. There could be a potential link between beneficial bacteria and a reduction in ASD symptoms. In this study, patients with ASD were classified as having mild or high-functioning ASD. The presence of these beneficial bacteria may contribute to a lower risk of developing more severe ASD symptoms.

This study also identified the differences in fungi and viruses as well as in microbial pathway in ASD, expanding beyond previous studies that focused primarily on bacteria [9]. With the advancement of metagenomic sequencing technologies, other microbial communities could also be analyzed in depth to provide more insight in this area. In the current study, the fungus *Mucor circinelloides* was found to be much more common in the ASD group. It is a naturally growing mold that can lead to potentially fatal infections in individuals with compromised immune systems [55]. Most viruses identified in this study, such as *Toutatisvirus toutatis* and *Aurodevirus hiberniae*, are bacteriophages associated with the gut microbiome that may interact with gut. Certain pathways were found to differ between the ASD and control groups. For example, the inosine-5′-phosphate biosynthesis III pathway was more prevalent in the ASD group. This pathway has also been found to be more abundant in the gut microbiota of smokers [56]. Our understanding of the relationship between the gut microbiota and ASD remains limited, and the functions of these microorganisms are not yet fully understood.

The observation of reduced bacterial diversity in the gut microbiota of individuals with ASD aligns with broader microbiome research, which often links lower microbial diversity to various health conditions. In this study, we found that individuals with ASD had variations in their microbial richness, with a significant correlation being observed between the diversity of bacterial species in the gut and the presence and intensity of autistic traits. This finding is consistent with studies on children with ASD [10,57] and suggests that bacterial metabolites may play a role in the development of ASD symptoms. The change in the microbial composition in persons with ASD is commonly referred to as “dysbiosis”, which is characterized by an imbalance in the microbiota along with alterations in its metabolic activities and functional composition [48]. While gut microbiota symbiosis supports the maintenance of normal host physiology [58], dysbiosis has been associated with various other conditions, including depression, anxiety, and Parkinson’s disease [59].

In our study, the DASH scores did not differ significantly between the ASD and control groups, although distinctive dietary patterns have been reported in individuals with ASD [60]. This discrepancy may reflect either reduced dietary variability within our sample or the limitations of the DASH score in capturing characteristic eating behaviors in ASD, such as selective eating and textural sensitivities. The relationship between diet, the gut microbiota, and ASD remains complex and elusive. It is still unclear whether alterations in the gut microbiota are a cause, a consequence, or both [61,62]. Supporting the potential influence of diet, a large metagenomic analysis of stool samples from individuals with ASD suggested that microbiome differences may mirror dietary preferences linked to core ASD features [63]. Conversely, evidence from a recent randomized, double-blind, placebo-controlled trial indicates that modulating the gut microbiota through oral fecal microbiota transplantation may lead to improvements in social behaviors, as reflected in changes in Vineland Adaptive Behavior Scale social domain scores [64]. Taken together, these findings highlight the need for further research—particularly longitudinal cohort studies—to better understand the dynamic relationships among diet, microbial composition, and ASD.

In this study, we developed prediction models to aid in the diagnosis of ASD in adults. The first model, incorporating gut microbiota data and Autism Spectrum Quotient questionnaire responses, achieved an AUC of 0.9551, while the second model, based solely on gut microbiota data, attained an AUC of 0.8076. Although ASD can be diagnosed in children as young as two years old, many individuals remain undiagnosed until adulthood [19]. These undiagnosed adults face increased risks of emotional and functional challenges due to exhibiting unrecognized ASD symptoms [19]. Currently, there is a lack of validated diagnostic tools for adults, as most of the tools are exclusively designed for children [65]. Diagnostic tools created for children are not always suitable for use in adults [66]. Although the Autism Spectrum Quotient can be a useful screening tool for identifying autistic traits in adults, it has several limitations, including its inappropriateness for individuals with low intellectual ability or limited reading comprehension [67]. Therefore, there is a critical need for objective assessment methods, such as biomarker-based tools, to support the diagnosis of ASD in adults. Various biomarkers have been explored for the diagnosis ASD, such as the C-reactive protein, oxytocin, iron, and zinc, but their accuracy is not ideal [68]. A recent study investigated gut microbiota markers as standalone diagnostic tools for ASD in children, without incorporating additional questionnaires, and achieved an AUC of 0.91 [15]. Our study extends this to the gut microbiota in adults, supporting the potential application of the gut microbiota as a diagnostic biomarker for high-functioning ASD. When used together, gut microbiota profiles and questionnaire responses may complement each other and further enhance diagnostic accuracy.

While this study sheds new light on the altered gut microbiota composition in adults with high-functioning ASD, there are some limitations. First, the sample size in this study was small, and the number of females in both the ASD and control groups was low, which limits the generalizability of the findings. Therefore, our results should be validated in larger and more gender-balanced samples of adults with ASD. Second, we did not evaluate our prediction model using samples from an independent cohort, and to our knowledge, there are no available public metagenomics sequencing data for adults with ASD. Finally, as this was a cross-sectional study, the causal relationship between the observed differences in gut microbiota composition and ASD cannot be established and verified. Future longitudinal studies are necessary to examine the lifetime trajectory of ASD and its interaction with the gut microbiota, and to determine whether microbial changes influence clinical symptoms over time.

## 5. Conclusions

In summary, our study reveals the altered gut microbiota composition in adults with high-functioning ASD and underscores the potential application of the gut microbiota as a non-invasive diagnostic tool. These findings have significant clinical implications for improving diagnostic processes to more effectively and efficiently identify high-functioning ASD in adults. Furthermore, they open avenues for developing potential intervention strategies, including gut microbiota modulation, to better support individuals with ASD.

## Figures and Tables

**Table 1:** Demographic information of the study population.

	ASD Group (n = 45)	Control Group (n = 45)	*p* Value
Age (years)			
Median (IQR)	23 (4)	25 (4)	0.004
Gender			
Male	41	40	1.000
Female	4	5	
Race			
Chinese	42	37	0.268
Malay	1	4	
Indian	2	2	
Others	0	2	
BMI (kg/m^2^)			
Mean (SD)	26.0 (8.77)	24.5 (6.23)	0.696
Housing type			
1–3 room government flat	5	2	0.466
4–5 room government flat	25	29	
private flat or landed property	15	14	

## Data Availability

The original contributions presented in this study are included in the article/Supplementary Material. Further inquiries can be directed to the corresponding authors.

## Supplementary Materials

The following supporting information can be downloaded at: https://www.mdpi.com/article/10.3390/nu17111748/s1. Figure S1. The correlations between Autism Spectrum Quotient scores and Chao1 Index values. *p* < 0.001 as measured by Pearson’s correlation test across both ASD and control groups. Figure S2. Volcano plot of differentially abundant bacterial species between ASD and control groups. Dots with yellow color represent the top 50 bacterial species with the largest log2 fold change. Figure S3. Volcano plot presenting differentially abundant fungal species between ASD and control groups. Dots with yellow color represent the fungal species with adjusted *p*-value < 0.05. Figure S4. Volcano plot presenting differentially abundant viral species between ASD and control groups. Dots with yellow color represent the viral species with adjusted *p*-value < 0.05. Figure S5. Volcano plot presenting differentially abundant pathways between ASD and control groups. Dots with yellow color represent the pathways with adjusted *p*-value < 0.05. Figure S6. The associations between number of features selected and mean AUC values. Figure S7. SHAP (SHapley Additive exPlanations) summary plot illustrating the impact of the selected features on the model’s classification output. Figure S8. Logistic regression performance analysis. The ROC curves were generated based on three datasets: gut microbiota data combined with Autism Spectrum Quotient questionnaire data (red curve), gut microbiota data alone (blue curve), and Autism Spectrum Quotient questionnaire data alone (yellow curve). Figure S9. Decision tree performance analysis. The ROC curves were generated based on three datasets: gut microbiota data combined with Autism Spectrum Quotient questionnaire data (red curve), gut microbiota data alone (blue curve), and Autism Spectrum Quotient questionnaire data alone (yellow curve). Figure S10. Naïve Bayes performance analysis. The ROC curves were generated based on three datasets: gut microbiota data combined with Autism Spectrum Quotient questionnaire data (red curve), gut microbiota data alone (blue curve), and Autism Spectrum Quotient questionnaire data alone (yellow curve). Table S1. List of features selected in the prediction model. Table S2. Summary of classification outcomes (number of true positives, true negatives, false positives, and false negatives) for each model.

## Abbreviations

The following abbreviations are used in this manuscript:

ASD	Autism spectrum disorder
ID	Intellectual disability
AND	Adult Neurodevelopmental Service
IMH	Institute of Mental Health
DSM-5	Diagnostic and Statistical Manual of Mental Disorders, Fifth Edition
SD	Standard Deviation
BMI	Body mass index
FGI	Functional Gastrointestinal
DASH	Dietary Approaches to Stop Hypertension
CPM	Copies per million
FDR	False discovery rate
ROC	Receiver operating characteristic
AUC	Area under the curve
IQR	Interquartile range
PCoA	Principal coordinate analysis
SCFA	Short-chain fatty acids
