# Supplementary material for: The Gut Microbiota in Young Adults with High-Functioning Autism Spectrum Disorder and Its Performance as Diagnostic Biomarkers
Source: Nutrients. 2025 May 22;17(11):1748. doi: 10.3390/nu17111748 (PMC12158130; doi:10.3390/nu17111748)
Supplement: Supplementary file 1 [file nutrients-17-01748-s001.zip › nutrients-3629487-supplementary.pdf]

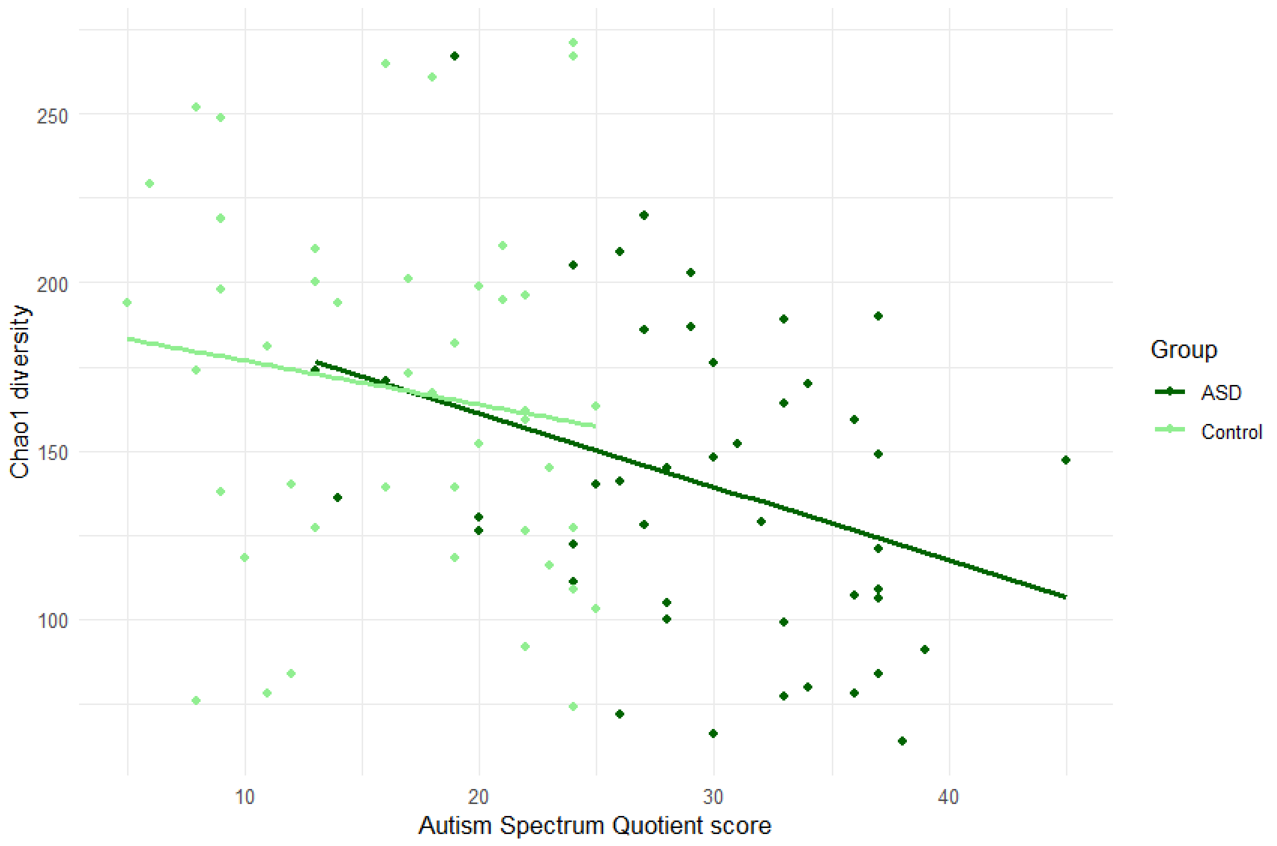

Figure S1. The correlations between Autism Spectrum Quotient scores and Chao1 Index values. P <0.001 as measured by Pearson's correlation test across both ASD and control groups.

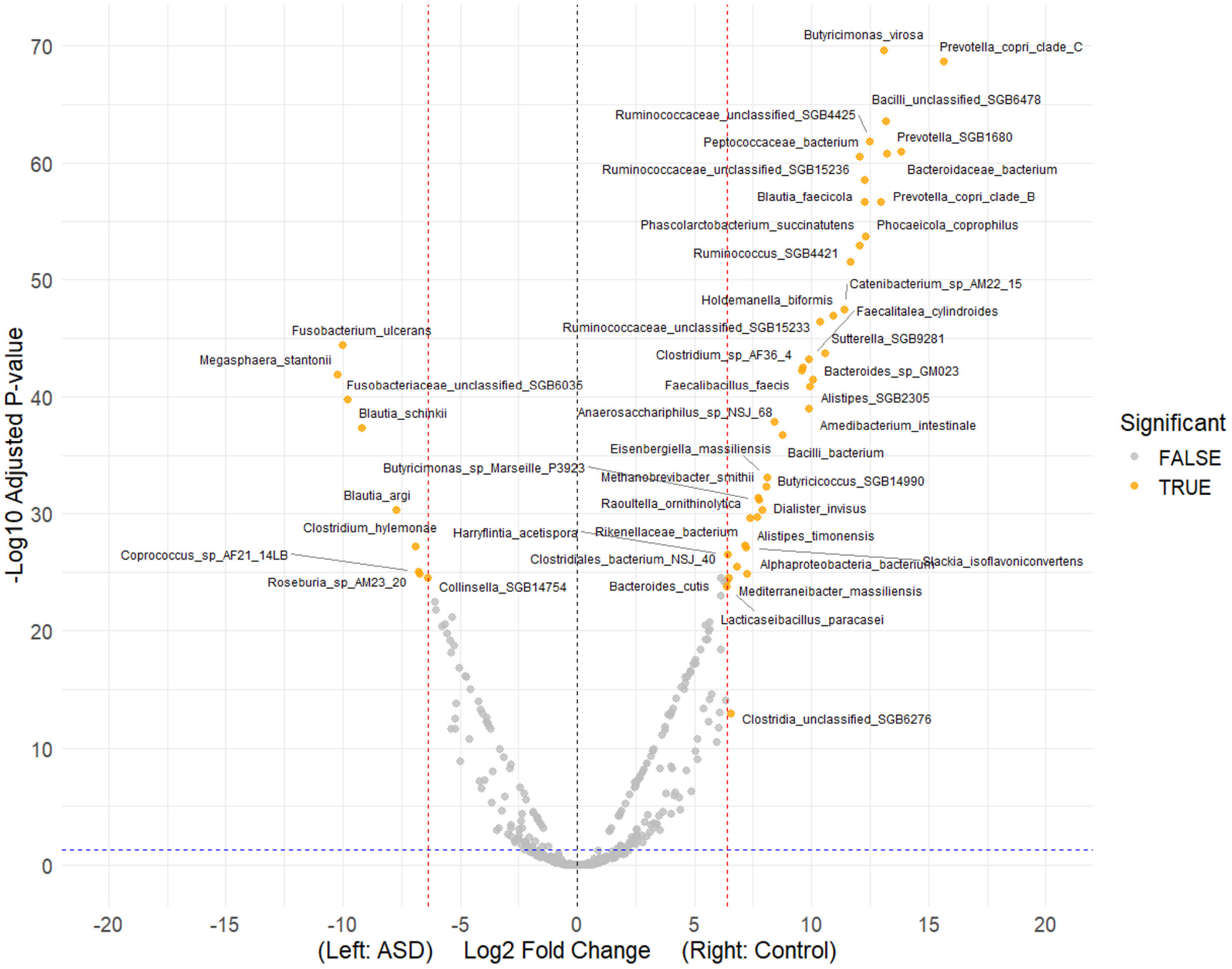

Figure S2. Volcano plot of differentially abundant bacterial species between ASD and control groups. Dots with yellow color represent the top 50 bacterial species with the largest log2 fold change.

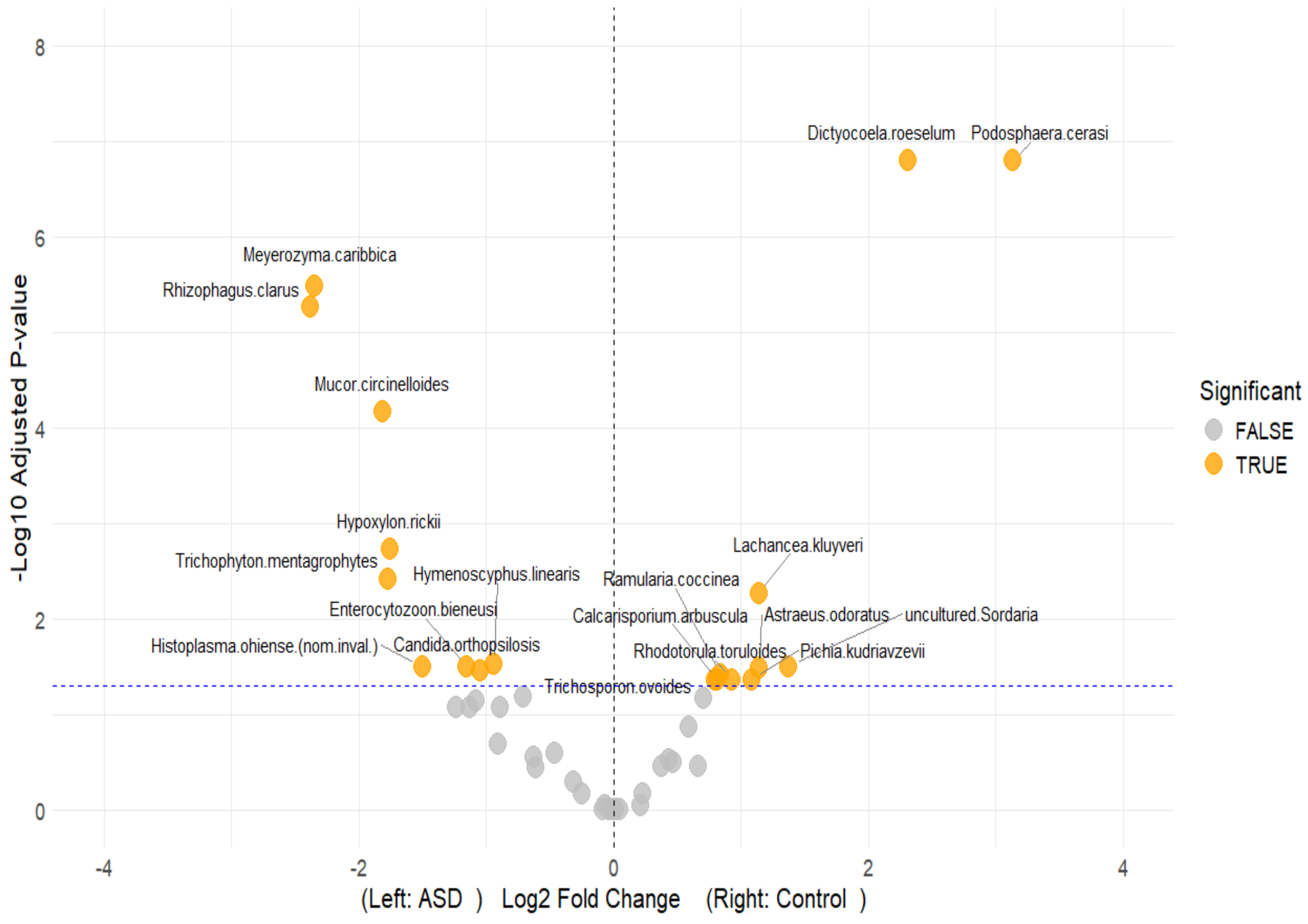

Figure S3. Volcano plot presenting differentially abundant fungal species between ASD and control groups. Dots with yellow color represent the fungal species with adjusted p-value < 0.05.

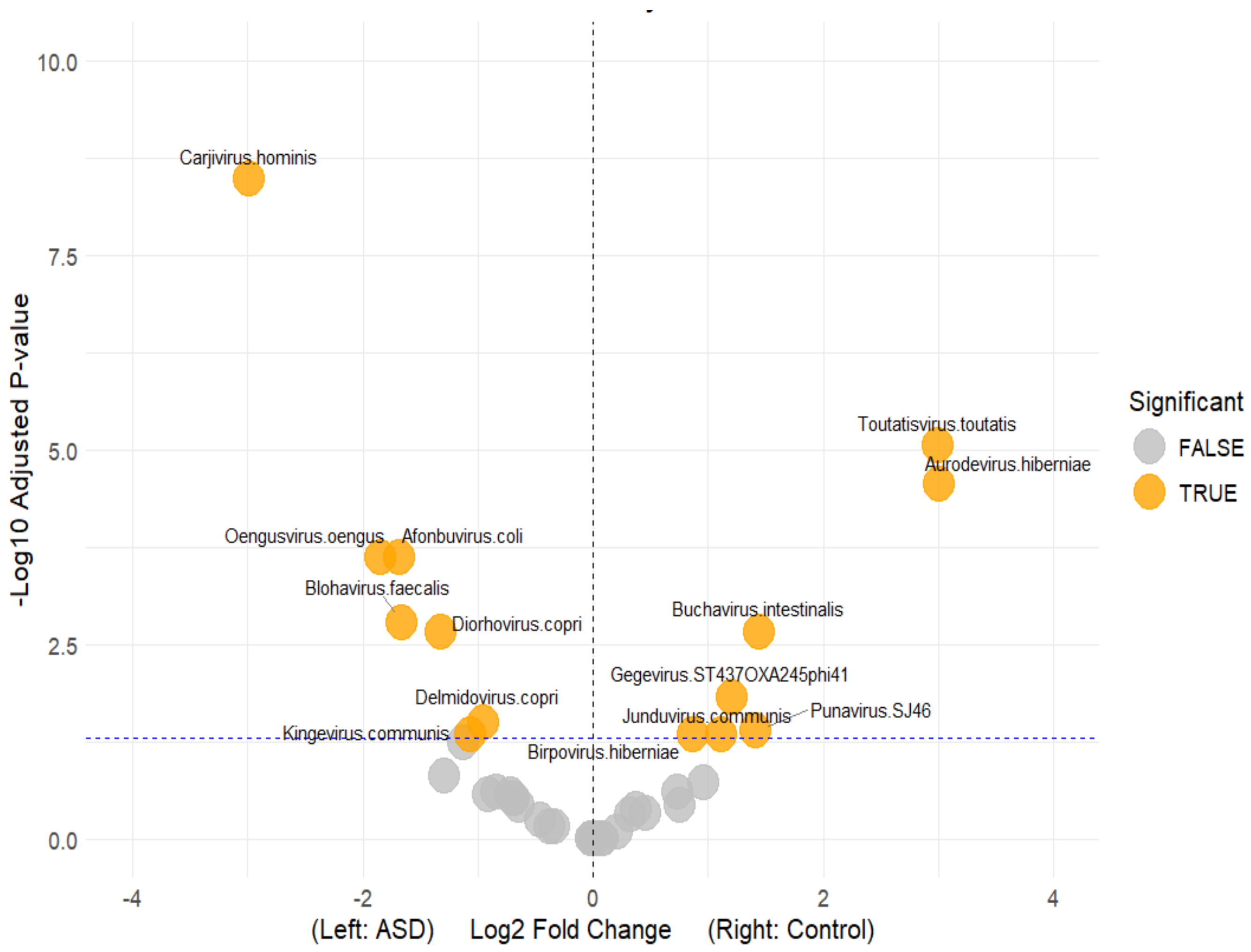

Figure S4. Volcano plot presenting differentially abundant viral species between ASD and control groups. Dots with yellow color represent the viral species with adjusted p-value < 0.05.

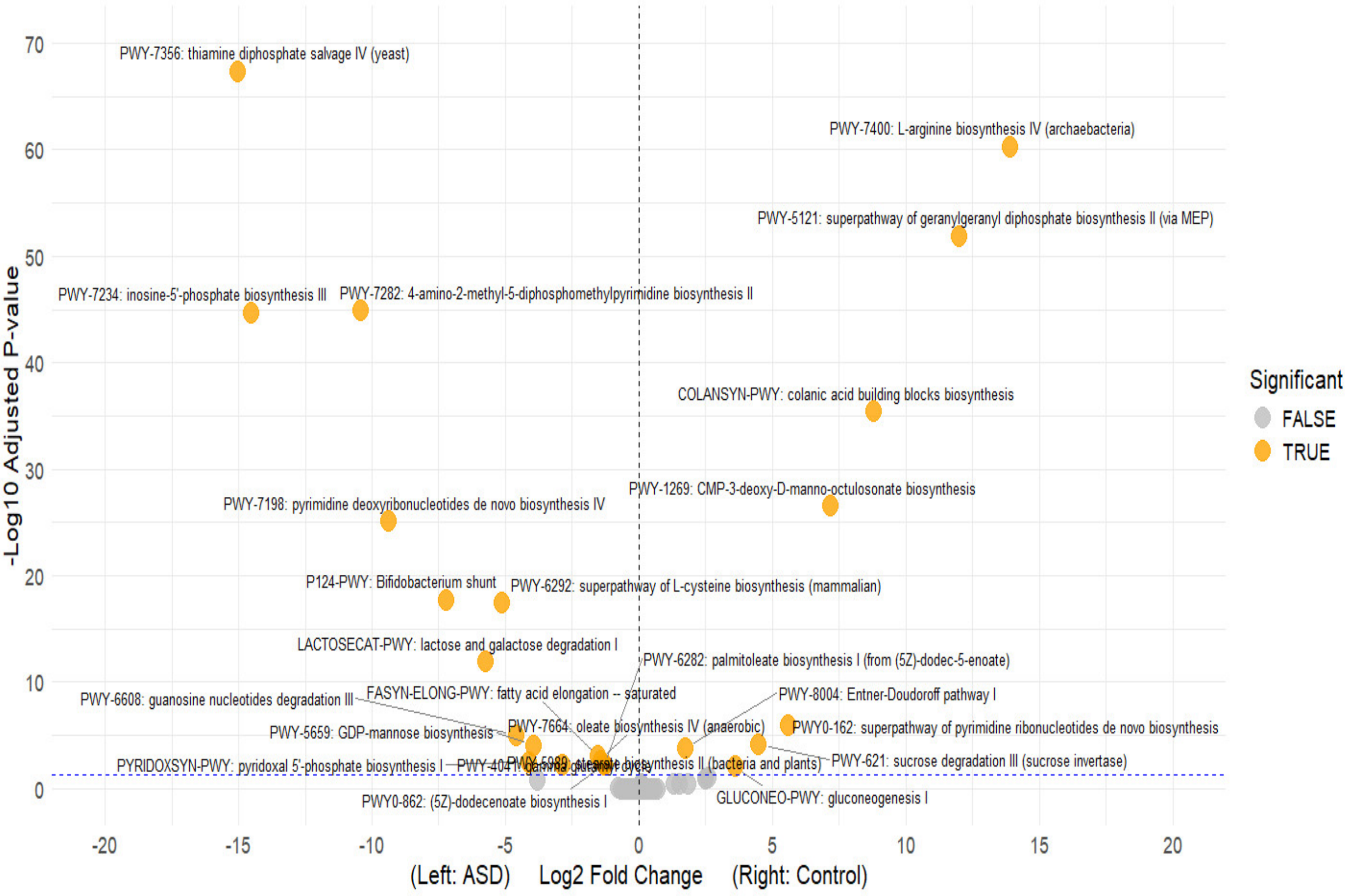

Figure S5. Volcano plot presenting differentially abundant pathways between ASD and control groups. Dots with yellow color represent the pathways with adjusted p-value < 0.05.

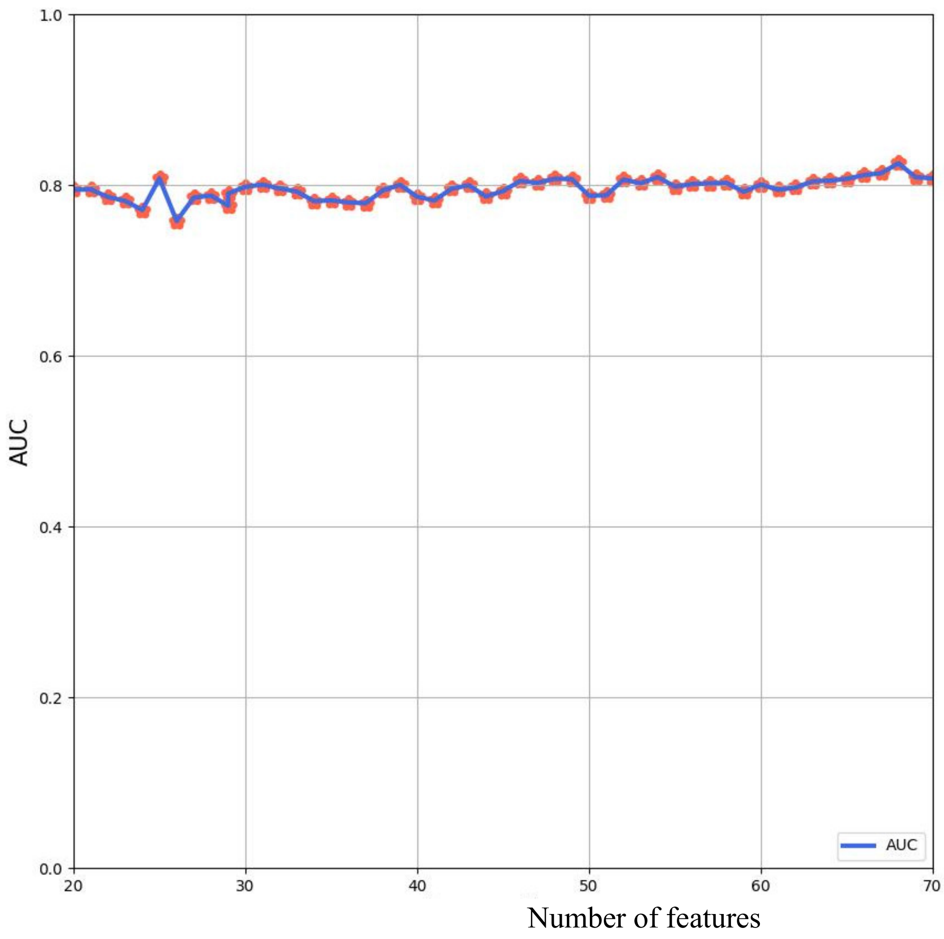

Figure S6. The associations between number of features selected and mean AUC values.

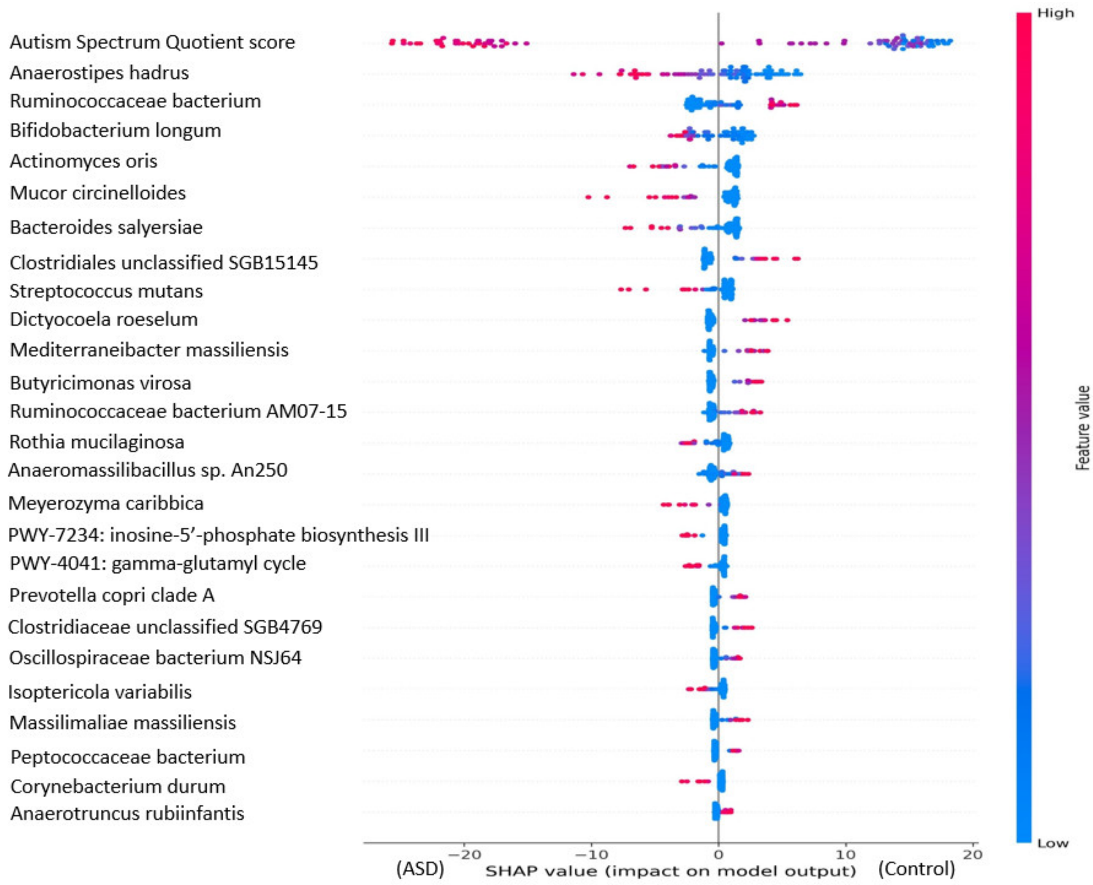

Figure S7. SHAP (SHapley Additive exPlanations) summary plot illustrating the impact of the selected features on the model's classification output.

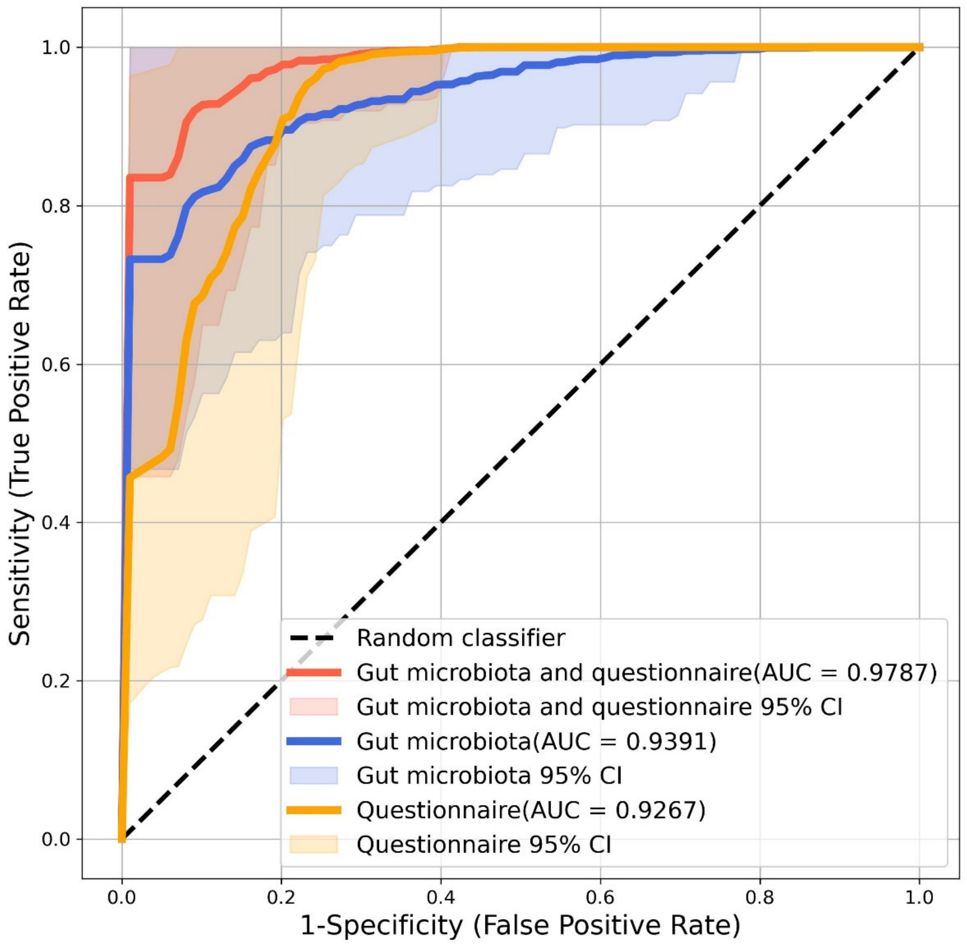

Figure S8. Logistic regression performance analysis. The ROC curves were generated based on three datasets: gut microbiota data combined with Autism Spectrum Quotient questionnaire data (red curve), gut microbiota data alone (blue curve), and Autism Spectrum Quotient questionnaire data alone (yellow curve).

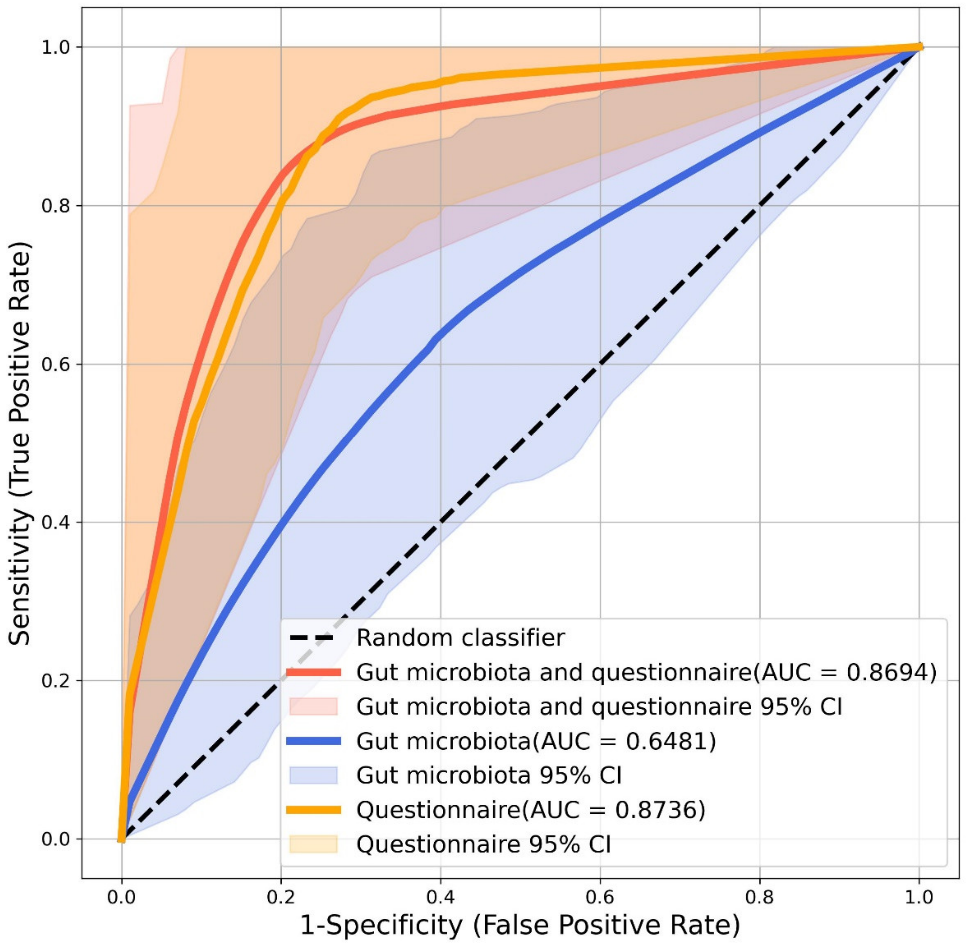

Figure S9. Decision tree performance analysis. The ROC curves were generated based on three datasets: gut microbiota data combined with Autism Spectrum Quotient questionnaire data (red curve), gut microbiota data alone (blue curve), and Autism Spectrum Quotient questionnaire data alone (yellow curve).

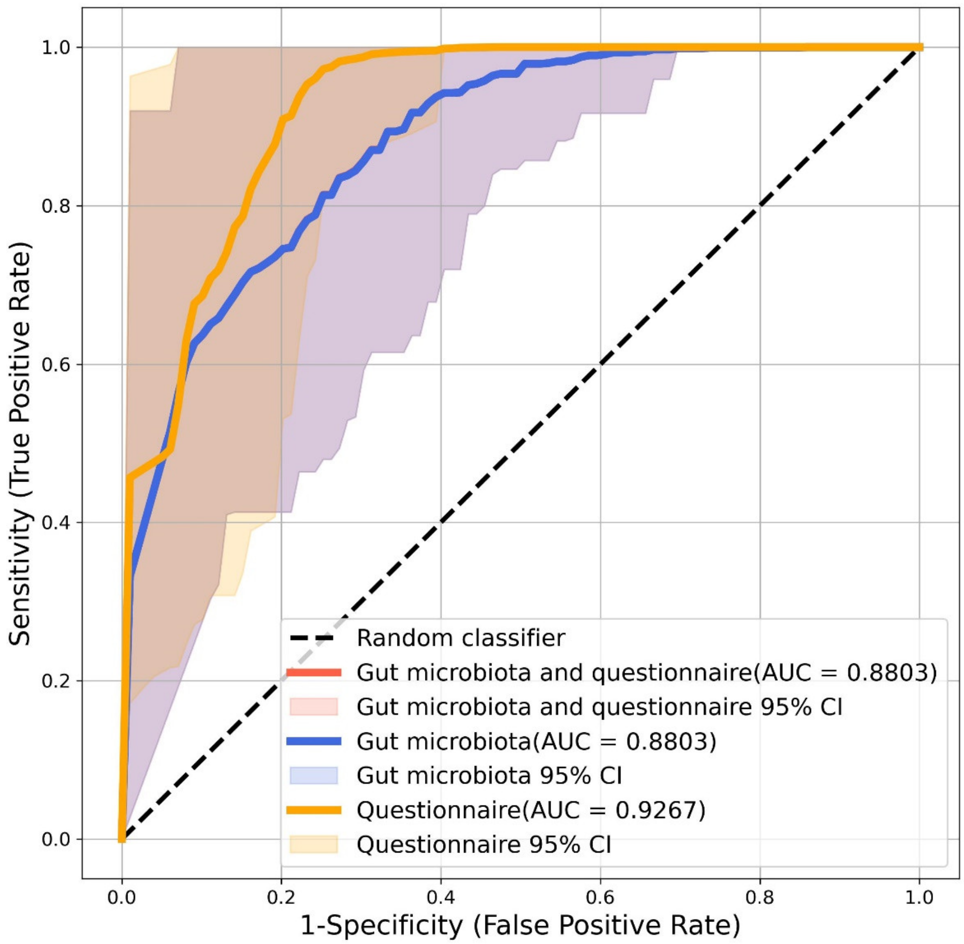

Figure S10. Naïve Bayes performance analysis. The ROC curves were generated based on three datasets: gut microbiota data combined with Autism Spectrum Quotient questionnaire data (red curve), gut microbiota data alone (blue curve), and Autism Spectrum Quotient questionnaire data alone (yellow curve).

Table S1. List of features selected in the prediction model

Group	Name of feature	Change in ASD
Bacteria	Anaerostipes hadrus	↑
	Actinomyces oris	↑
	Bifidobacterium longum	↑
	Bacteroides salyersiae	↑
	Corynebacterium durum	↑
	Isoptericola variabilis	↑
	Rothia mucilaginosa	↑
	Streptococcus mutans	↑
	Anaerotruncus rubiinfantis	↓
	Anaeromassilibacillus sp. An250	↓
	Butyricimonas virosa	↓
	Clostridiales unclassified SGB15145	↓
	Clostridiaceae unclassified SGB4769	↓
	Mediterraneibacter massiliensis	↓
	Massilimaliae massiliensis	↓
	Oscillospiraceae bacterium NSJ64	↓
	Ruminococcaceae bacterium AM07-15	↓
	Ruminococcaceae bacterium	↓
	Peptococcaceae bacterium	↓
	Prevotella copri clade A	↓
Fungi	Meyerozyma caribbica	↑
	Mucor circinelloides	↑

	Dictyocoela roeselum	↓
Pathways	PWY-4041: gamma-glutamyl cycle	↑
	PWY-7234: inosine-5’-phosphate biosynthesis III	↑

Table S2. Summary of classification outcomes (number of true positives, true negatives, false positives, and false negatives) for each model

Model	True Positives	True Negatives	False Positives	False Negatives
Based on gut microbiota	36	35	10	9
Based on Autism Spectrum Quotient questionnaire	45	35	10	0
Based on gut microbiota and Autism Spectrum Quotient questionnaire	45	34	11	0
